# Risk Assessment of Healthcare Workers at the Frontline against COVID-19

**DOI:** 10.12669/pjms.36.COVID19-S4.2790

**Published:** 2020-05

**Authors:** Saqib Ali, Sara Noreen, Imran Farooq, Amr Bugshan, Fahim Vohra

**Affiliations:** 1 Saqib Ali Department of Biomedical Dental Sciences, College of Dentistry, Imam Abdulrahman Bin Faisal University, Dammam, Saudi Arabia; 2 Sara Noreen Department of Medicine, Khyber Teaching Hospital, Peshawar, Pakistan; 3 Imran Farooq Department of Biomedical Dental Sciences, College of Dentistry, Imam Abdulrahman Bin Faisal University, Dammam, Saudi Arabia; 4 Amr Bugshan Department of Biomedical Dental Sciences, College of Dentistry, Imam Abdulrahman Bin Faisal University, Dammam, Saudi Arabia; 5 Prof. Fahim Vohra Department of Prosthetic Dental Science, College of Dentistry, King Saud University, Riyadh 11545, Saudi Arabia

**Keywords:** COVID-19, Healthcare, Mortality Rate, Personal Protective Equipment, Risk

## Abstract

The novel coronavirus disease 2019 (COVID-19) is a global pandemic. Healthcare workers (HCWs) are on the frontline of treating patients infected with COVID-19. However, data related to its infection rate among HCWs are limited. The aim was to present evidence associated with the number of HCWs being infected with COVID-19 from most viral affected countries (Italy, China, United States, Spain, and France). Furthermore, we looked into the reasons for HCWs COVID 19 infections and strategies to overcome this problem. Early available evidence suggested that HCWs are being increasingly infected with the novel infection ranging from 15% to 18% and in some cases up to 20% of the infected population. Major factors for infection among HCWs include lack of understanding of the disease, inadequate use and availability of Personal Protective Equipment (PPE), uncertain diagnostic criteria, unavailability of diagnostic tests and psychological stress. Therefore the protection of HCWs by authorities should be prioritized through education and training, the readiness of staff, incentives, availability of PPEs, and psychological support.

## INTRODUCTION

The novel corona virus disease 2019 (COVID-19) outbreak rapidly spread throughout the world causing a public health crisis globally.^1^ The first case of COVID-19 emerged in December, 2019 in Wuhan, China and recently World Health Organization declared it a pandemic.^2^ The symptoms for the Severe Acute Respiratory Syndrome (SARS)-COV 2 virus related COVID-19 infection include fever, dry cough, shortness of breath, and pneumonia that could be mild to severe in intensity.^3^ It is believed that the incubation period of this virus is 2-14 days and symptoms usually appear between these days.^4^ It is reported that the disease spreads through direct contact or by inhaling respiratory droplets or airborne aerosols from the infected patients,^5^ and data from China indicates that around 85% of human-to-human transmission occurs in a family groups.^6^ Infected individuals in multiple instances act as asymptomatic carriers, as they do not show the signs and symptoms of COVID-19. As a result, the most critical measure in protection and prevention of spread is “hand washing” and “social distancing”.^7^

Looking back into history, it would not be wrong to say that infectious diseases have killed more individuals than any war.^8^ The growing number of cases of COVID-19 pose a major threat to international health and wellbeing of humans.^9^ Healthcare workers (HCWs) are always on the frontline, whether it is an elective treatment, a medical emergency, or dealing with a pandemic like COVID-19.^10^ This makes HCWs at the greatest risk of getting exposed to infection.^11^ The aim of this communication is to review the number of HCWs being infected with COVID-19 from countries with the highest mortality rate (as of 31^st^ March, 2020). Furthermore, we looked into the reasons behind HCWs getting infected and strategies to overcome this problem. The Google Scholar, Web of Science and the PubMed database were searched with combination of keywords including, “COVID-19”, “COVID-19 and HCWs”, and “COVID-19 and cross-infection” and “SARS-COV 2, Health care, Risk, Mortality rate and Personal Protective Equipment (PPE). Twenty-eight studies and news articles were included, data extraction was performed and a summary is presented.

## HEALTHCARE WORKERS INFECTED WITH COVID-19

Reports of HCWs, infected with COVID-19 are emerging globally.^12^ It is imperative to prevent COVID-19 infections of HCWs by taking appropriate measures as higher infection rates for HCWs from different countries are showing increased risk. A summary of HCWs getting infected in five countries with highest mortality rate of COVID-19 is presented below.

### COVID-19 affecting HCWs in Italy

Italy is one of the top five countries globally, which has been most affected by COVID-19 and has the highest mortality rate globally.^12^ According to a report published by International Council of Nurses, COVID-19 infection among nurses in Italy makes up 9% of the total cases.^13^ Moreover, data analysis also revealed that 20% of all HCWs dealing with COVID-19 patients were positively infected.^14^

### COVID-19 affecting HCWs in China

China was the first country to report the spread of COVID-19 infections.^2^ Studies have suggested that nearly 3,300 HCWs have been infected, with 22 suffering from severe respiratory distress leading to death (Feb, 2020).^14^ A recent study from Wuhan has linked proximity to COVID-19 patients, long duty hours, and suboptimal hand hygiene as a possible risk factors for HCWs infection with COVID-19.^15^

### COVID-19 affecting HCWs in Spain

Early published literature on Spanish HCWs infection of COVID-19 indicates higher numbers with rapid spread. It is reported that a staggering 9400 HCWs have been infected (March 2020) with COVID-19, which is nearly 15% of all early infected cases in Spain.^16^ These numbers are astonishingly high and if not prevented could result in a collapse of the health care system.

### COVID-19 affecting HCWs in the United States (U.S.)

The exact number of HCWs infected with COVID-19 is not known at present, however a report from *The Washington Post* narrated that “dozens of HCWs have been infected already and more are now quarantined after the exposure”.^17^ In addition, figures released by the state of Ohio reported, 18% HCWs with positive COVID-19 infections. Furthermore, in the state of Minnesota, it was stated that, every one-in-five diagnosed case was a HCW.^18^ Looking at the rising numbers of HCW infections, multiple health care societies have raised concern for their protection and prevention. A board member of American College of Emergency Physician has expressed his concern that ‘*this is just the tip of the iceberg’*.^17^

### COVID-19 affecting HCWs in France

The overall rate of infections in France is among the highest globally, however, data reporting related to HCWs who got infected with COVID-19 is limited. The Public Hospital System in France has reported 490 HCWs being infected among more than 100,000 workers.^19^ Unfortunately; these numbers are projected to rise in the coming weeks.

From the above-mentioned data and reports, it is clear that HCWs are being increasingly infected with COVID-19. This makes a double negative impact on the healthcare system globally, as the system is burdened by the high patient number and infection among HCWs aggravates the already existing shortage of people working to curb the spread of the disease.

### COVID 19 transmission to Healthcare Workers (HCWs)

**Fig.1:**
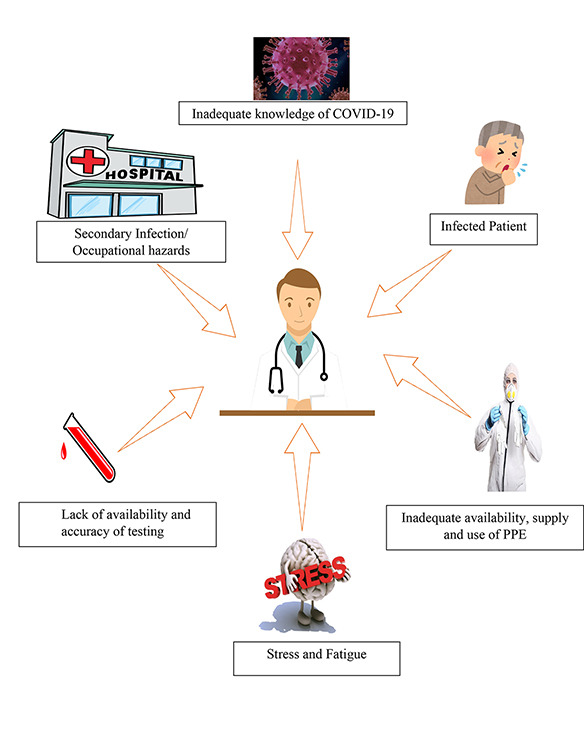
Factors in transmission of COVID-19 to HCWs.

In this time of COVID 19 pandemic, hospitals are flooded with infected symptomatic and asymptomatic patients. HCWs are at the forefront of the treatment and management of COVID-19 infections, working in close proximity to the infectious virus. Besides, the unpreparedness of the healthcare system and the novelty of the SARS-COV2 infection has made the HCWs a common and easy target for these infections. One of the most important routes of secondary transmission of COVID-19 is the spread through a hospital setting. A primary reason for HCWs to get infected is the insufficient accurate scientific data on SARS-COV 2 including, its virulence factors, survival outside a host, resistant strains, incubation period and infection pathophysiology. Therefore, resulting in infections to HCWs and from HCWs to individuals.^20^ Reports from the early viral spread in the Chinese city of Wuhan have suggested that a high number of HCWs, who were unaware of the transmissibility and severity of COVID 19, got infected while treating the infected patients.^20^ This was primarily due to the lack of information of the disease and its viral spread. In addition, the preventive measure for COVID 19 infections require specialized personal protective equipment (PPE) like respirators, N-95 masks, non-perforated gowns and visors or face shields for protection from infections. Due to the large scale of infections globally, the supply of these necessary PPEs has been irregular to say the least.^21^ Moreover, most of the PPEs are non-reusable and should be discarded with the utmost precautions to prevent transmission. Therefore the inadequate availability and improper use of PPEs is a critical contributing factor in the high COVID 19 infection rates of HCWs. The understanding of the diagnostic criteria and diagnostic test of COVID-19 infection has developed over a period of months. The RT-PCR testing for the identification of virus is not readily available at all health care facilities and for all suspected patients. In addition, the test results on average are available in more than 24 hours. Therefore the difficulty and lack in widespread reliable testing and the uncertainty of the diagnostic criteria is also associated with the transmission of infection to HCWs.^20^ Likewise, stressful working environment, long working hours leading to fatigue and isolation related psychological issues also contribute to increased probability for HCWs infection of COVID-19.^22^ Some other factors which can predispose HCWs to infection could possibly be inadequately cleaned and sanitized hospital surfaces, compromise in disinfection of medical equipment and lack of training and education related to the viral pandemic.^23^ An illustration of the common factors in the transmission of infection to HCWs is presented in Fig.1.

### Strategies to prevent COVID-19 among healthcare workers

The health regulatory bodies should start with training and education of medical and supporting staff including nurses, technicians, dentists, doctors, paramedics, receptionists and cleaners through online mandatory courses according to the updated protocols as issued by WHO and Centre for disease control (CDC) in order to protect HCWs from hospital-acquired COVID-19 infection.^24^,^25^ The education should include, information on the type of virus, its transmission, disease signs and symptoms, diagnostic criteria, vulnerable patient groups and treatment and management protocols. Hospital personnel training should include the type of PPEs, their proper use, cleaning, re-use and disposal; and the doctor and patient hygiene. In addition, guidelines for all specialities of healthcare providers must be issued to protect individuals and prevent transmission of COVID 19 infection to doctors, operators and patients.^21^ Moreover, standard cleaning and disinfection measures for individuals and premises should be performed religiously to further prevent the spread of the virus and minimize the risk of cross infection.^26^

A critical aspect is the protection of the HCWs who are at risk, including individuals more than 50 years old those with presence of a systemic disease, smokers and disable individuals.^27^ These HCWs should be identified and not assigned duties in wards and premises managing COVID 19 patients. These individuals can be assigned to non-patient care and administrative teams. To protect the HCWs and their families, staff should undergo routine medical checks, including temperature checks and RT-PCR test. In addition, HCWs should isolate themselves in hospital provided residence and maintain social distancing from family members and other staff. To prevent psychological distress and manage stressful conditions, psychological evaluations, and counseling sessions should be available for vulnerable staff. To preserve mental wellbeing, HCWs should practice healthy eating, physical activity, minimum 6-8 hours of sleep and communication with family and friends.

According to Daily DAWN of May 4, 2020, the number of infected cases in Pakistan till May 3, 2020 were 20,130 with 459 confirmed deaths. The number of healthcare professionals who got infected were four hundred fifty.. Looking at the trends worldwide, it can be assumed that COVID 19 infection among HCWs is a major threat to the individuals and the overall task force for COVID 19 infections in the country. A quick spike of infections among HCWs could result in higher morbidity and mortality rates. A combination of this healthcare predicament with the financial crisis would push the country into a downward spiral. Therefore protection of HCWs should be the priority of the government’s health regulatory bodies in Pakistan and worldwide. As a high number of HCWs practicing strict cross-infection controls and implementation of evidence based decisions are integral to the fight against COVID-19.

## CONCLUSION

Healthcare workers are at the front line of defence against COVID-19 infection among communities. However early evidence suggests that HCWs are being increasingly infected with COVID-19 ranging from 15% to 18% and in some cases up to 20% of the infected population. Major factors for COVID 19 infection among HCWs include lack of understanding of the infection, inadequate use and availability of PPE, uncertain diagnostic criteria and unavailability of the diagnostic test; and psychological stress. Therefore it is recommended, that protection of HCWs by authorities should be prioritized through education and training, the readiness of staff, incentives, availability of PPEs and psychological support.

### Authors’ Contributions

**SA:** Idea, literature review, manuscript writing.

**SN:** Literature review, manuscript writing.

**IF:** Literature review, manuscript writing.

**AB:** Literature review, manuscript writing.

**FV:** Study conception, manuscript writing, review and final approval of the manuscript. He is also responsible for the integrity of the study.
